# Changes in grassland management and linear infrastructures associated to the decline of an endangered bird population

**DOI:** 10.1038/s41598-020-72154-9

**Published:** 2020-09-16

**Authors:** Ana Teresa Marques, Francisco Moreira, Rita Alcazar, Ana Delgado, Carlos Godinho, Hugo Sampaio, Pedro Rocha, Nuno Sequeira, Jorge M. Palmeirim, João Paulo Silva

**Affiliations:** 1 cE3c – Centro de Ecologia, Evolução E Alterações Ambientais, Faculdade de Ciências da Universidade de Lisboa, Edifício C2, Campo Grande, 1749-016 Lisbon, Portugal; 2 CIBIO/InBIO, Centro de Investigação Em Biodiversidade E Recursos Genéticos, Laboratório Associado, Universidade Do Porto, Campus Agrário de Vairão, 4485-661 Vairão, Portugal; 3 CIBIO/InBIO, Centro de Investigação Em Biodiversidade E Recursos Genéticos, Laboratório Associado, Instituto Superior de Agronomia, Universidade de Lisboa, Tapada da Ajuda, 1349-017 Lisbon, Portugal; 4 LPN – Liga para a Proteção da Natureza, Centro de Educação Ambiental de Vale Gonçalinho, 7780-909 Castro Verde, Portugal; 5 CEABN/InBIO – Centro de Ecologia Aplicada “Professor Baeta Neves”, Instituto Superior de Agronomia, Universidade de Lisboa, Tapada da Ajuda, 1349-017 Lisbon, Portugal; 6 MED – Instituto Mediterrâneo para a Agricultura, Ambiente e Desenvolvimento, LabOr – Laboratório de Ornitologia, Universidade de Évora, Polo da Mitra, 7002-774 Évora, Portugal; 7 SPEA – Sociedade Portuguesa para o Estudo das Aves, 1070-062 Lisbon, Portugal; 8 ICNF/PNVG – Instituto de Conservação da Natureza E Florestas, Parque Natural Do Vale Do Guadiana, 7750-350 Mértola, Portugal; 9 QUERCUS – Associação Nacional de Conservação da Natureza, Parque Florestal de Monsanto, 1500-045 Lisbon, Portugal

**Keywords:** Ecology, Zoology

## Abstract

European grassland birds are experiencing major population declines, mainly due to changes in farmland management. We analyzed the role of habitat availability, grazing management and linear infrastructures (roads and power lines) in explaining spatial and temporal variation in the population density of little bustards (*Tetrax tetrax*) in Portugal, during a decade in which the species population size halved. We used data from 51 areas (totaling ca. 1,50,000 ha) that were sampled in two different periods (2003–2006 and 2016). In 2003–2006, when the species occurred at high densities, habitat availability was the only factor affecting spatial variation in bustard density. In the 2016 survey, variation in density was explained by habitat availability and livestock management, with reduced bird numbers in areas with higher proportions of cattle. Population declines across the study period were steeper in areas that initially held higher densities of bustards and in areas with a higher proportion of cattle in the total stocking rate. Areas with higher densities of power lines also registered greater density declines, probably due to avoidance behavior and to increased mortality. Overall, our results show little bustards are currently lacking high quality grassland habitat, whose persistence depends on extensive grazing regimes and low linear infrastructure densities.

## Introduction

Natural and semi-natural grasslands across the world are known for their high biodiversity value^1–3^. Extensively managed High Nature Value (HNV) farmlands are recognized as key habitats for several taxa in Europe, namely butterflies^4^ and birds^5,6^, and the maintenance of such landscapes is considered a priority for European biodiversity^7^. However, they are often subjected to changes in management, such as conversion to other land uses, agriculture intensification or abandonment^3^. Intensification of management is usually associated to an increase in the use of agrochemicals or in livestock density^8,9^. Often, these changes are supported by targeted policies. For example, in Europe, changes in livestock management are promoted by the Common Agricultural Policy (CAP) through coupled payments (per livestock head), leading to an increase in livestock numbers, mainly cattle^10,11^.

Grasslands or pseudosteppes of the Iberian Peninsula are considered key farmlands habitats for European biodiversity, mainly due to their importance for threatened grassland bird species^12–15^. The traditional management of these systems created a heterogeneous landscape due to the extensive cultivation of dry cereal crops in rotation with pastures to support sheep raising, which produced a patchy system of cereal fields, stubbles, fallow lands, plowed lands and pastures that supported a widely rich and variable grassland bird community, including several species with unfavorable conservation status at the European and world level such as great bustard (*Otis tarda*), Montagu’s harrier (*Circus pygargus*) or little bustard (*Tetrax tetrax*)^14,16^. Despite the ecological importance of these HNV systems, so far EU policies have failed to maintain these landscapes. In fact, such systems are being rapidly replaced by systems specialized on cattle and consequently increasing the proportion of pastures in the landscape^10,11^. Even though these changes maintain grasslands, they potentially reduce the quality of the habitat for biodiversity by reducing landscape heterogeneity^17^, and promoting changes in sward structure and crop management, e.g. hay harvesting times^18–20^.

In addition to grassland management changes, infrastructures are also known to affect grassland birds. Infrastructures are major drivers of human-related effects in the Anthropocene, associated with habitat changes (e.g. habitat loss and fragmentation), behavioral effects (avoidance and displacement due to disturbance) and mortality of numerous animal taxa^21,22^. Roads and power lines are known to cause high levels of mortality of bird species^23^, and population effects have already been described in raptors^24^, bustards^25^ and owls^26^, among other bird groups. Regarding grassland birds, several studies report high mortality levels due to collision, mainly with power lines, and a displacement effect related with human structures, as power lines, roads and wind farms, has also been described^27–30^.

The little bustard is a Near Threatened ground-nesting grassland bird^31^ and considered a bioindicator species of grassland quality in southern Europe^32^. In Iberia, which holds ca. 95% of the population of the species in Western Europe (Iñigo and Barov, 2010) the species depends principally on grasslands to breed^34–36^. A network of Special Protection Areas (SPA) holding well conserved grasslands were designated for the protection of this and other steppe species. Despite that, little bustard numbers dropped dramatically during the twenty-first century; a mean population decline of 49% was recorded in Portugal between 2003–2006 and 2016^37^ and a decline of 48% was recorded in Spain for the same period^38^. Habitat loss due to agricultural intensification, leading to reductions in fallow land area, were associated with declines and local extinctions of the little bustard in other parts of Europe^33,39^. However, evidence collected so far suggests that the declines in the Iberian Peninsula may not be fully explained by such drivers. In Portugal, for example, the decline in male densities was stronger within SPAs than in unprotected areas, even though the amount of grassland habitat did not change markedly within protected areas^37^. This indicates that a reduction in the amount of grassland habitat is not the only driver of the population decline, and that a deterioration of the quality of this habitat, due to changes in management, is also likely to be involved^37,38^. Collision with power lines is the main anthropogenic cause of mortality of the little bustard, affecting 3.4–3.8% of adult birds per year^40^. This is the highest mortality rate per collision with power lines ever recorded for a species. Morphological characteristics and bird sensorial perception are key species-specific features that explain such a high mortality rate^41–44^, but behavioural changes (as flocking behaviour or flight height) across seasons were also considered relevant^45,46^. In addition to mortality, little bustards also avoid the vicinity of anthropogenic infrastructures, mainly to roads and power lines^47–52^. Therefore, such structures are likely to influence little bustard spatial distribution and population density.

In this study, we used the results of two countrywide surveys of little-bustard (2003–2006 and 2016) in Portugal to analyze the drivers of spatial and temporal variation in little bustard densities at a landscape level. Bird data was gathered through a standardized field protocol applied across the Iberia Peninsula since 2003 that targets male birds, as females have a cryptic behavior and are hard to survey, due to their morphological characteristics and behavior. We specifically tested the potential roles of (1) the availability of the grassland habitat, i.e. habitat quantity; (2) livestock density as a proxy of habitat quality and (3) the amount of linear infrastructures, in explaining spatial and temporal changes in the density of the species in the two study periods.

## Results

### Major changes between surveys

**Figure 1:**
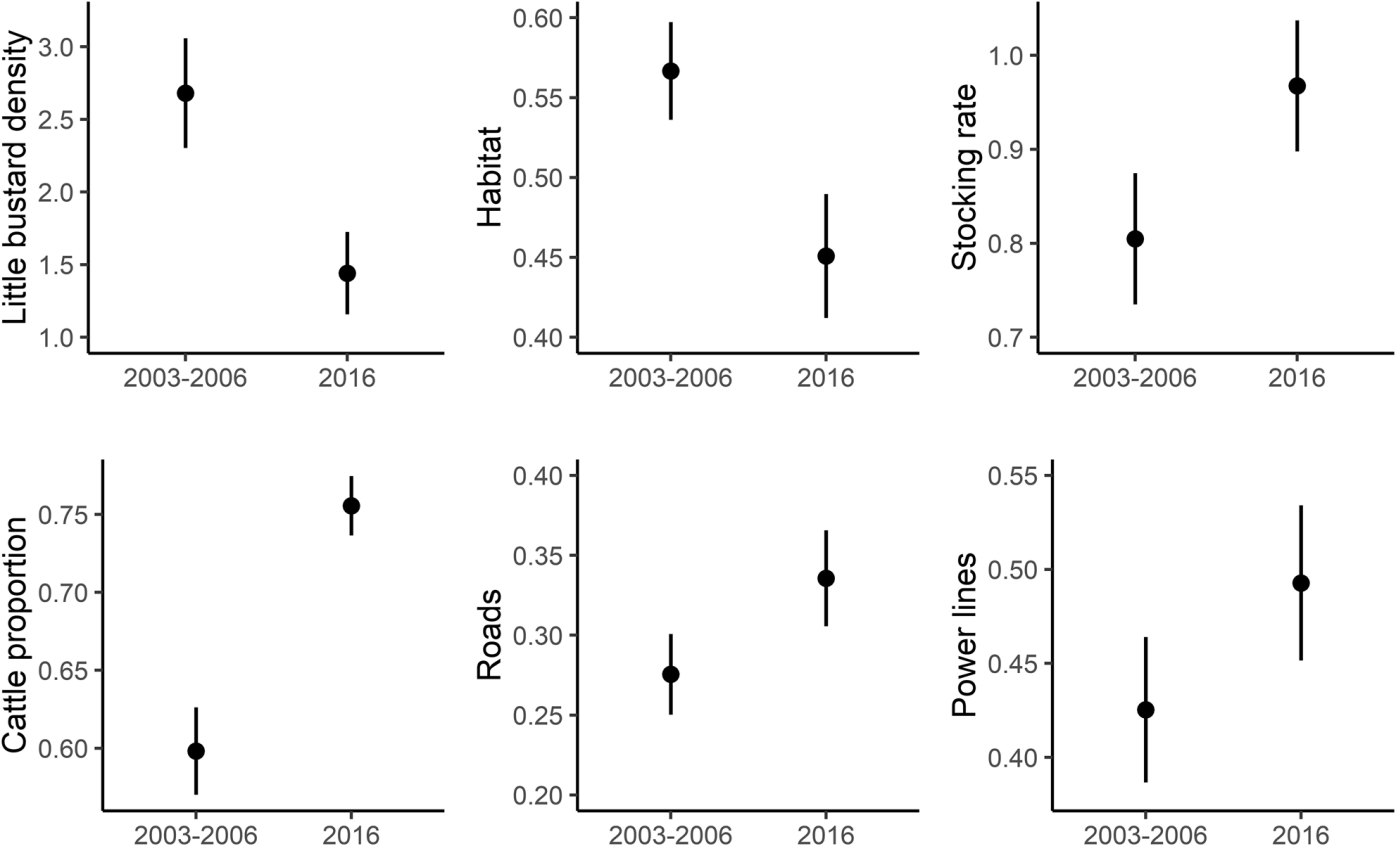
Variation in little bustard density and environmental predictors (mean and standard errors) between the two surveys periods (2003–2006 and 2016). The grazing regime predictors (stocking rate and cattle proportion) are presented for 1999 and 2009, the only periods with livestock statistics available for the study area.

The mean little bustard density significantly declined from 2.68 ± 0.38 males/ha in 2003–2006 to 1.44 ± 0.28 males / ha in 2016 (Table S1, Fig. 1). Most of the sampled areas (n = 35 out of 51 study areas) showed a negative trend between the two surveys and the species disappeared in 12 of them.

Habitat availability, i.e. the proportion of grassland area within the surveyed areas, significantly declined from 0.57 ± 0.03 to 0.45 ± 0.04 (Table S1, Fig. 1), with declines in 28 of the sampled areas. Most of these areas lost over 5% of grassland habitats (n = 19), 6 of which with losses between 40 and 89%.

There was a significant increase of the estimated stocking rate and the proportion of cattle in the total stocking rate experienced a significant increase (Table S1, Fig. 1), across the whole region. A significant increase of the linear infrastructures also occurred across the study period (Table S1, Fig. 1), road density went from 0.27 km/km^2^ ± 0.03 to 0.33 ± 0.03 km/km^2^, and power line density from 0.43 km/km^2^ ± 0.04 to 0.49 ± 0.04 km/km^2^.

### Drivers of spatial variation in little bustard densities for each period

**Figure 2:**
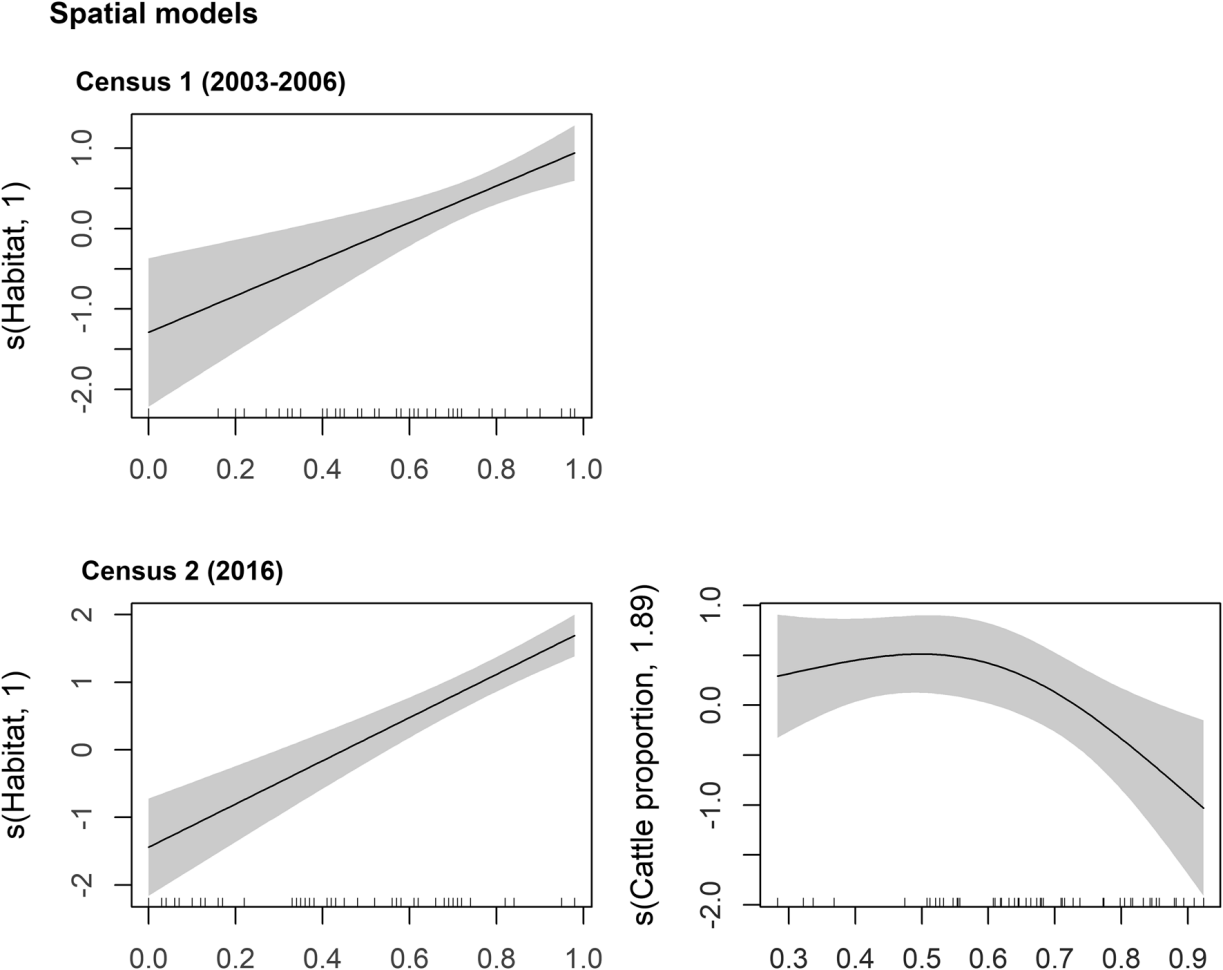
Generalized additive model partial effects for the two spatial models of the relationship between the little bustard density in each survey (2003–2006 and 2016) and the environmental predictors. Shaded areas represent 95% confidence intervals. The y-axis shows the contribution of the fitted centered smooth terms s (names of the predictor, estimated degrees of freedom) to the response variable (little bustard density in each survey). Ticks on the x-axis represent the location of observations along the predictor.

In the first survey (2003–2006), habitat availability was the only significant predictor of spatial variation in little bustard densities, with higher densities occurring in areas with higher proportions of grasslands. In the 2016 survey, habitat availability was also important, but the model also included a variable related to grazing management, i.e. the species was more abundant in areas with lower proportion of cattle in the total stocking rate (< 60%) (Table 1 and Fig. 2). This latter model had a higher explanatory power compared to the former one (71.2% vs. 24.6%). There was no significant autocorrelation in both model residuals (Figs. S1 and S2).

**Table 1:** Summary statistics for the three GAM models: the two spatial models tested the effect of the environmental predictors on little bustard density in each survey period (2003–2006 and 2016) and the population variation model tested the effect of the environmental predictors on the delta in little bustard density across surveys (2016–2003–2006).

	Model coefficients	Estimate	SE	t	edf	F	*p* value	Deviance explained (%)
**Spatial models**								
Survey 2003–2006 Density	Intercept	0.87	0.16	5.49			0.000	24.6
	Habitat				1.00	14.82	0.000	
Survey 2016 Density	Intercept	− 0.09	0.18	− 0.47			0.643	71.2
	Habitat				1.00	55.94	0.000	
	Cattle proportion				1.88	5.42	0.007	
**Population variation model**								
Density variation	Intercept	− 1.24	0.14	− 8.73			0.000	80.6
	Density survey 2003–2006				1.76	85.81	0.000	
	Habitat_mean				1.84	21.41	0.000	
	Cattle proportion_mean				1.82	9.47	0.001	
	Power lines_mean				1.00	5.17	0.028	

*SE* Standard error, *t* T statistics, *edf* estimated degrees of freedom, *F* F statistics.

### Drivers of little bustard temporal density variation

**Figure 3:**
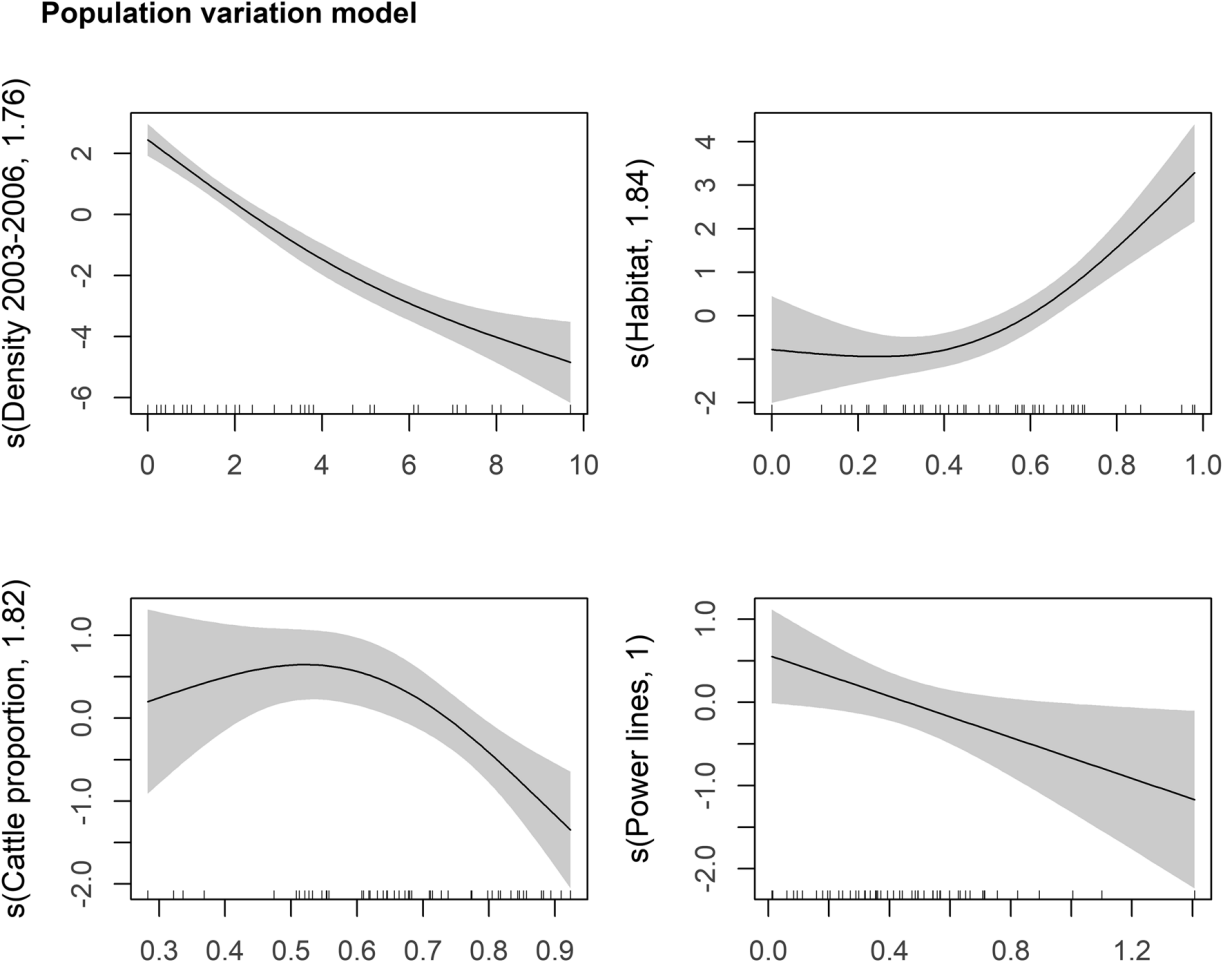
Generalized additive model partial effects for the population variation model of the relationship between the delta in little bustard density across surveys (2016–2003–2006) and the environmental predictors. Shaded areas represent 95% confidence intervals. The y-axis shows the contribution of the fitted centered smooth terms s (names of the predictor, estimated degrees of freedom) to the response variable (delta in little bustard density between survey). Ticks on the x-axis represent the location of observations along the predictor.

As for the population variation model (Table 1 and Fig. 3), larger declines in little bustard density occurred in areas with higher densities of bustards in the first survey, a larger proportion of cattle in the stocking rate and a higher density of power lines. Areas with a high proportion of available habitat (> 40%) were the ones with smaller losses. There was no significant autocorrelation in model residuals (Fig. S3).

## Discussion

### Changes in bustard densities and their drivers across time

The little bustard is considered a bioindicator species of HVN farmlands supporting a high diversity community of birds in Iberia^32,39^. Contrary to other ground-nesting birds, such as great bustard, Montagu’s harrier or stone curlew (*Burhinus oedicnemus*), the little bustard is subject to coordinated monitoring programs across the Iberian Peninsula, which makes it a good indicator of the overall conservation status of the agricultural landscapes within Iberia. In this study, we recorded a 46.3% variation of little bustard male density in our study areas, from 2003–2006 to 2016, matching the patterns previously reported for the whole region^37^. Although large scale monitoring programs of little bustard only collect data on male birds (as females have a cryptic and secretive behavior that makes it difficult to study them), studies conducted so far suggest that a sex-ratio biased towards males may be an issue in areas of intensive agriculture^53,54^, whilst in extensive and large grasslands areas the sex-ratio seems to be balanced^55^.

Changes in key population drivers likely explain the observed trends. First, there was a reduction of available habitat across time. The major losses were observed in areas allocated to recent irrigation projects, which converted grasslands to permanent crops such as olive groves or vineyards. In fact, the whole Alentejo region is facing major landscape changes during the twenty-first century, as large expanses of open agricultural lands have been transformed into permanent crops. As a result, the area devoted to annual crops decreased 66%, while permanent crops increased by 23% between 2000 and 2016 (Fig. S4^56^). These changes were mostly located outside SPA, in accordance with the findings of recent studies^37,57^ that also reported habitat loss outside these protected areas, in contrast with a greater habitat persistence within SPA. Our results are also consistent with those found in Spain, where declines in little bustard density are correlated to habitat loss, and fallow land in particular^39^.

As for habitat quality, the remaining grasslands likely degraded over time due to different reasons: (1) from 2000 to 2016 the number of beef cattle increased by 48% in the Alentejo region, while sheep declined by 27% (Fig. S4^56^), leading to the observed overall increase of the stocking rate along with the increased proportion of cattle, with expected negative impacts on vegetation structure and nest trampling and desctruction^58,59^. (2) A consequence of the increased numbers of cattle is that the area devoted to hay production has been increasing in the region to ensure the increased fodder demands^19^, a land use that did not occur in the traditional system based on rotational cereal crop. This increase of hay production has likely consequences for the bustard population as the mowing of hay fields is carried out over one month earlier, compared to the traditional system, and coinciding with the beginning of egg hatching leading to nest destruction and increasing adult mortality, primarily females^19^. (3) Changes in livestock management also represent the loss of the traditional rotation system based on cereal production and its resulting heterogeneous landscape, which is replaced by permanent pastures of lower quality for ground nesting birds (Fig. S4^56^). The intensification of land use and livestock management were promoted by the CAP reform of 2003^10,11^. It should be noted, however, that the available data relating to livestock management used in this study was not detailed enough to fully characterize the livestock density and management in the study area, hence future studies should focus on providing further details on the effect of grazing and variable stocking rate levels on ground nesting birds. (4) Finally, there was an overall increase of anthropogenic infrastructures (namely roads and power lines), which are known to negatively impact little bustards. Collisions with power lines are a major source of mortality for the species in the Iberia Peninsula^40^ and power lines and roads are also a known source of displacement effects for the species^47–52^.

### Drivers of spatial variation in little bustard densities

In 2003–2006, higher population densities of bustards were found in areas with higher habitat availability and this was the only significant driver of spatial variation in bustard densities. This shows that habitat availability had a very strong influence on population size, as it promoted a disproportionate increase in bustard densities, providing evidence that habitat availability was also the single best indicator of habitat quality. This effect can also be explained by conspecific attraction that is known to occur in grassland species^60,61^, including in the little bustard^62^. In 2016, habitat availability was still relevant, but livestock management also became significant explaining spatial variation in male densities. In fact, following the overall increase of livestock density, the bustards occurred in higher densities in areas with lower (< 60%) proportions of cattle (compared to sheep) (Fig. 2). Previous studies found that intermediate levels of cattle grazing density were preferred by little bustards^54,63^ and the highest densities of the species in Alentejo (in 2007–2008) were recorded in areas grazed by cattle with seasonal rotational pastures^36,55^. However, in our study, the increased proportion of cattle, also associated with higher stocking rates (Pearson r = 0.40, *p* = 0.004), seems to have led to the avoidance of some areas by the bustards in 2016. The reduced little bustard density in areas with higher proportions of cattle suggests that grassland management associated with cattle cause a decrease in habitat quality, when compared with similar stocking rate values for sheep.

### Drivers of temporal changes in population densities

In spite of the clear trend for population density declines over time, the regional variation in population densities during the ca. 10-year period were quite variable across areas (ranging from losses of 6.9 males/ha to gains of 2.9 males/ha), thereby suggesting that the prevalence of driving forces behind such changes varied across areas.

Major declines in density (absolute value) were observed in areas where the bustards occurred in higher densities during the first survey. These high densities are likely a good indicator of overall habitat quality for the species^36^, coinciding with areas where the magnitude of the impacts of habitat loss and degradation, as well as the overall population decline, are more clearly expressed. The loss of male densities in remaining areas was limited by their already low densities.

Habitat quantity was also relevant to attenuate the decline of the species between 2003–2006 to 2016, as study areas with the largest expanses of grasslands (> 40% of the study area surface) were the ones with smaller density losses (some even with gains). Large and continuous grasslands are known to host high density of little bustards in Portugal^35,36^, and our results suggest that such landscapes have more resilient populations, which are able to buffer the general population decline. Little bustards preference for extensive and open landscapes is probably due to their exploded lek mating system, as larger fields (> 50 ha) allow greater aggregations of males, which are preferred and are visited more frequently by females, with lower disturbance levels, higher proportion of nesting females and lower rates of predation as a result of reduced edge effects^36^.

Areas dominated by cattle were the ones with higher reductions of little bustard density between surveys. This suggests that higher cattle densities and respective changes in grassland management are causing changes in the quality of grassland habitats (see previous sections).

Areas with a higher density of power lines suffered greater density losses between surveys. Power lines are known to affect the spatial distribution of the little bustard, although it is not clear if the causal factor is an increased perceived predation risk (as power line poles are frequently used as perches by little bustard predators, such as large raptors) or neophobia^52,64^. Additionally, power lines represent a major cause of anthropogenic mortality for the species^40^, with high fatalities due to collisions with power line cables known to occur at the onset of the breeding season^45^. These mechanisms may explain the reduction of male density in areas with more power lines.

The fact that the road network was not relevant in explaining spatial variation in the distribution of little bustards was unexpected, as roads are responsible for habitat fragmentation and a source of disturbance^65^, previously reported to affect the spatial distribution of the little bustard during the breeding season^36,47–49,51^. The lack of a statistically significant effect of road density on little bustard may be explained by the fact that both structures tend to occur spatially clustered, with power lines and roads running parallel, and we had a moderate positive correlation between these predictors in our dataset (Pearson r = 0.39, *p* = 0.004).

### Implications for conservation

Our work shows that both habitat quantity and quality are key for the little bustard. Conservation actions should focus principally on (1) avoiding further habitat losses, mainly due to the plantation of permanent crops (i.e. olive groves, vineyards or almond orchards); (2) avoid the expansion of linear infrastructures and mitigate the impacts of either existing and future structures, e.g. with the use of wire marking devices; and (3) reduce the cattle levels. Although we acknowledge that cattle livestock plays an important role to maintain the grassland habitat in Iberia, and some grassland bird species may even benefit from such livestock systems, current cattle stocking rates in Alentejo are not compatible with the conservation of the little bustard. This also highlights that management guidelines should take into account the historical background of each system, respecting the singularities and biodiversity value of each location, as general policies will not adjust for every farmland system^66^. To reduce the dominance of cattle in Iberian grasslands we suggest: (a) the total decoupling of cattle in the next CAP reform, so that farmers are paid per unit area with a fixed maximum cattle density, rather than a payment per cattle head; and (b) using the same mechanism to promote sheep grazing at moderate densities, so that these are not totally replaced by cattle. Reduced cattle densities will imply lower hay needs, with direct benefits for little bustards. The proposed recommendations aimed to manage the little bustard will also be beneficial to the overall grassland bird community, as high cattle densities likely affect other ground-nesting birds in our study area, including high conservation concern species as the great bustards or the Montagu’s harrier, mainly because of trampling and haying activities during the nesting period.

The decline of the little bustard in Alentejo is probably not only a result of pressures occurring during the breeding season. In fact, multiple threats, such as loss of post-breeding and wintering areas, high anthropogenic mortality and global warming^40,67,68^, may be acting synergistically. Little bustards are short-distance migrants^69,70^ that move towards more productive agriculture areas in northern, coastal or higher-altitude locations in Iberia during the dry summer season, in search of green vegetation^69,71^. Hence, the patterns we are witnessing at breeding areas may also be a consequence of the still unknown drivers occurring elsewhere. Regular monitoring programs would be important for a comprehensive understanding of the overall bustard decline and to understand the effect of land use shifts and other drivers of the current population trend. These programs should be implemented at different stages of the little bustard annual cycle, namely in the post-breeding and wintering areas (much less known that the breeding areas), and including a focus on the threats to the species during these seasons.

Overall, a successful recovery of little bustard populations will depend, not only on a better management of breeding habitat, but also on wider conservation actions during the remaining annual cycle that promote the reduction of anthropogenic mortality levels and maintain favorable non-breeding habitat.

## Methods

### Little bustard data

**Figure 4:**
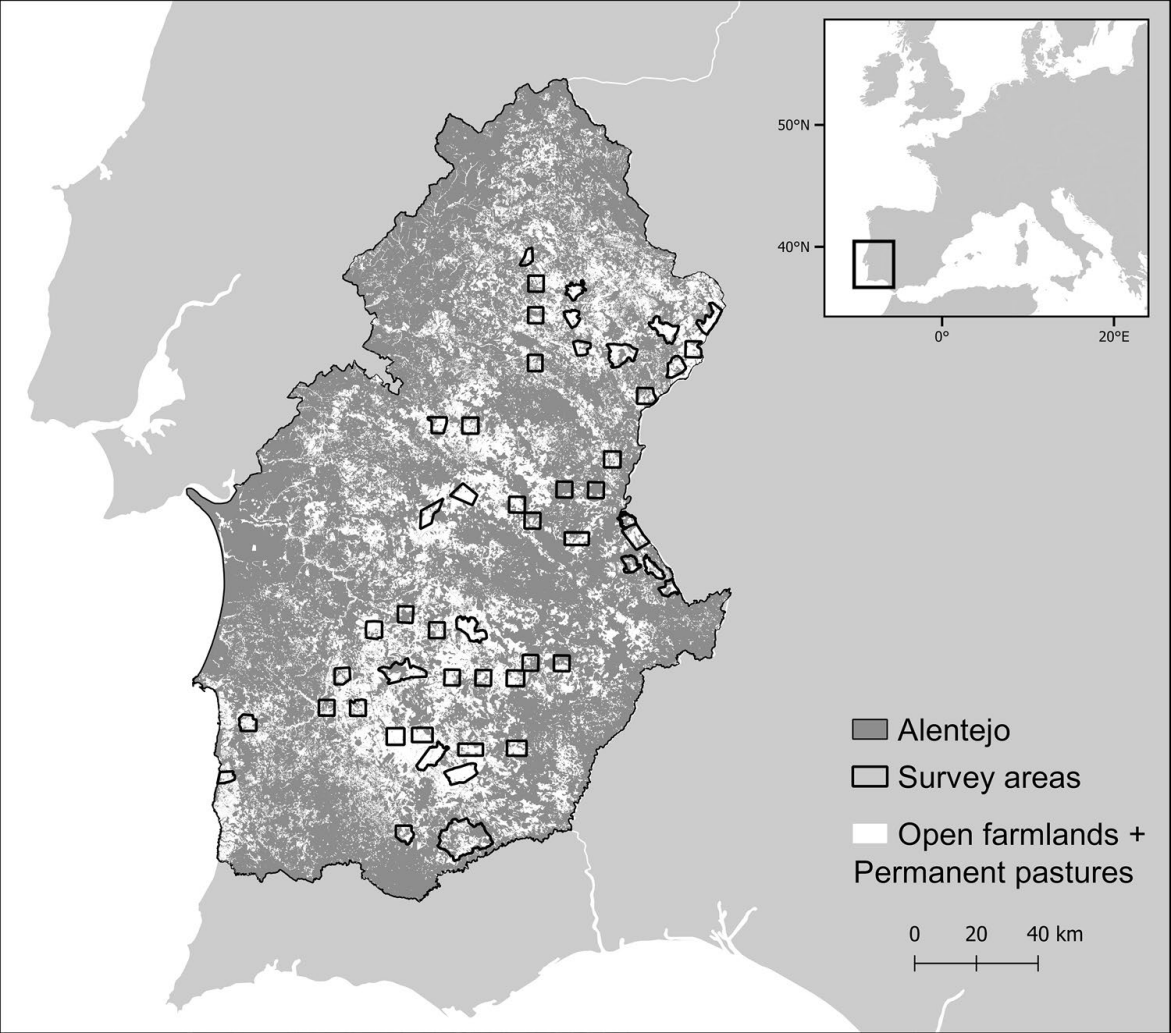
Location of the 51 survey areas in Alentejo, Portugal, and open habitats in the region (open farmlands and permanent pastures, based on the official land cover maps of mainland Portugal for 2007 and 2015^75^, publicly available on-line at https://mapas.dgterritorio.pt/geoportal/catalogo.html). Map created using the Free and Open Source QGIS 2.14.20^88^ by A.T.M.

Little bustard male densities were surveyed in two different time periods, 2003–2006 and 2016^37^, across 51 areas (totaling ca. 1,50,000 ha; mean = 2,889 ha; range = 1,657–9,997 ha) located in the Alentejo region, southern Portugal (Fig. 4). The region is home to the large majority of the national breeding population of the species^72^. Bird density was estimated following a standardized protocol targeting male birds, as females have a cryptic behavior and are hard to detect^34,73,74^. A network of point counts defined along non-paved roads, 600 m from each other and from paved roads or inhabited houses, was used to survey birds in each area, covering an average density of approximately 1.0 points/km^2^ per survey (range 0.47–3.00). In all 51 areas, 1,526 and 1,441 survey points were sampled in 2003–2006 and in 2016, respectively (differences due to changes in dirty road availability, as the access to some properties were closed by the land owners). Still, 99% of all sampling points were replicated in the exact same location of the previous survey. At each point location, little bustard males were counted within a 250 m radius during 5 min within the first three hours after dawn or three hours before dusk, during April and May. For further details on little bustard surveys see Moreira et al.^35^ and Silva et al.^37^.

### Potential drivers of bustard population density

The analysis was focused on a regional and landscape scale, hence, for each survey period, and for each of the 51 areas, information on four major types of potential drivers of spatial and temporal changes in little bustard densities were gathered (Table 2):

**Table 2:** Description and summary statistics for the predictor variables used to model little bustard density during the breeding season in Alentejo, Portugal.

Variable	Description	Mean (SD)	Range
Habitat	Proportion of the survey area covered with potential breeding habitat: non-irrigated annual crops, permanent pastures and fallow land	0.51 (0.25)	0–0.98
Stocking rate	Density of cattle and sheep livestock units per area of pastures and fallow land (LU/ha)	0.89 (0.59)	0.21–3.00
Cattle proportion	Proportion of cattle in the stocking rate	0.68 (0.19)	0–0.95
Roads	Density of roads in each survey area. The length of the structures at the survey area boundaries was divided in half (km/km^2^)	0.31 (0.19)	0–0.70
Power lines	Density of power lines in each survey area (km/km^2^)	0.46 (0.28)	0.01–1.41

Means, standard deviation, and range are provided.

#### Habitat availability

We quantified the amount of permanent pastures, non-irrigated annual crops and fallow land, the major land use types considered suitable for the species^35,36,46^, in each area. Land use data was collected from the official land cover maps of mainland Portugal for 2007 and 2015^75^, publicly available on-line at https://mapas.dgterritorio.pt/geoportal/catalogo.html. We used land use classes 2.1.1 and 2.3.1 as our cartographic base for non-irrigated annual crops (including fallow land) and permanent pastures, respectively. This information was later refined to match the surveys years based on (1) visual inspection of Google Earth and Bing images and (2) field validation. The proportion of the total surface of each area covered by suitable habitat was estimated (Table 2).

#### Grazing management

Agricultural statistics were used to characterize livestock densities in our survey areas, focused on the two main grazers of the region: cattle and sheep. Two variables were estimated: (1) stocking rate, i.e. the number of livestock units (LU) per area of pastures and fallow land (stocking rates were calculated according to the following ratio: cattle = 1 LU; adult sheep = 0.15 LU^76^), and (2) the proportion of cattle in the total (cattle + sheep) stocking rate (Table 2). Both variables were derived from the results of the national agrarian census (RGA—Recenseamento Geral Agrícola) of 1999 and 2009, complemented with information obtained from Instituto Nacional de Estatística regarding the amount of pastures and fallow land^76,77^. We used the smallest administrative region in the country (i.e. Freguesia) as our unit, and applied a weighted mean based on the area occupied by each Freguesia in our individual survey areas to obtain an estimate for each area. Due to the temporal lag between the little bustards’ surveys and the available data on livestock we used the mean value between 1999 and 2009 data as a proxy of average grazing intensity in our sampled areas. Some obtained values of estimated livestock densities were considered artificially high (in 3 of the sampled areas, with estimated values often one order of magnitude higher), as they do not represent the real density of livestock in the field and are probably related to animals in stables or declared by farmers but grazing in farms outside the region. So, we set densities to a maximum of 3 LU/ha, corresponding to the highest values estimated by direct counts in the field.

#### Linear infrastructures

We gathered data on the distribution of paved roads and power lines in both surveys and calculated their density (km/km^2^) per study area (Table 2). We used the data from Open-StreetMap contributors, namely the classes: motorway, trunk, primary, and secondary^78^, to identify the main paved roads. For power lines, we mapped both the transmission (> 110 kV) and the distribution (< 110 kV) networks, based on data provided by the electric companies in Portugal (REN and EDP). Both data were validated for each surveys period based on Google Earth, Bing images and field checks. We considered that roads bordering our study areas had a reduced effect when compared to the ones traversing them, as only one side of these structures is expected to directly affect the bustard populations within area boundaries. Therefore, the length of the roads at the boundaries of the survey areas was down weighted when calculating its density, by dividing the length in half.

### Data analysis

First, we fitted univariate Generalized Linear Mixed Models (GLMM) to test if little bustard density and potential drivers (habitat, stocking rate, cattle proportion, roads and power lines) varied across surveys, using the surveys year as a fixed factor and the sampled area as a random effect. These models were fitted in R^79^ with packages lme4^80^ and lmerTest^81^.

To access the main drivers influencing the density of the little bustard across the study region, in each survey, we first performed two models that assessed the factors influencing spatial variation in bird densities separately for each survey date (hereafter referred to as spatial models). These models included the density in each survey area as the response variable, and the predictors included: breeding habitat availability, stocking rate, cattle proportion, roads and power lines (Table 2). In each spatial model values for predictors corresponded to the sampled year, except for the two grazing predictors, where we used the mean value of the available data as described above. A third model, the population variation model, assessed the factors underlying spatial patterns of changes in the little bustard density. In this model the variation in bird density across time (survey 2016–survey 2003/2006) in each area was the response variable. As predictors, we included the mean value across surveys of breeding habitat availability, stocking rate, cattle proportion, roads, and power lines (Table 2), which aimed to access the main global pressures in each survey area, as values of these predictors across time were highly positively correlated (Spearman's rho = 0.74. 0.81, 0.85, 0.85 and 0.94, respectively, *p* < 0.001; Fig. S5). Additionally, we included the little bustard density in the first surveys as a predictor, as the magnitude of the absolute variation in density is obviously constrained by the initial value in the area.

We used Spearman correlation coefficient and variance inflation factors to check for collinearity between the explanatory variables (Zuur et al.^83^). Variance inflation factor values (all < 2.0) and pairwise correlations between explanatory variables (all |r|< 0.55) were low for our dataset, so all variables were used in the analysis.

Generalized Additive Models (GAM) were used to fit the three models, thereby accounting for potential non-linear responses^82,83^. The spatial models were fitted using a Gaussian distribution and a logarithmic link function, ensuring that fitted values were positive. To model the variation on the density values across surveys we used a Gaussian distribution with an identity link function. For the three models the optimal smoothing parameter was estimated by restricted maximum likelihood estimation (REML), and a basis dimension (k = 3) was defined to allow some complexity in the functions, while avoiding over-fitting the data. The models were fitted in R^79^ with the package mgcv^84^.

The modelling procedure involved the fitting of the full model, followed by backward elimination of non-significant (*p* > 0.05) variables to find the optimal model. The final model adequacy was evaluated by plotting residuals versus fitted values and explanatory variables, and the model fit was evaluated by the proportion of the null deviance explained (Zuur et al.^83^). Spline correlogram plots with 95% pointwise confidence intervals calculated with 1,000 bootstrap resamples were used to check for spatial autocorrelation in model residuals^85^. We assumed that variable selection and parameter estimation were unbiased if there was no significant autocorrelation in model residuals^86^. Correlograms were estimated in R with the ncf package^87^.

## Supplementary information

Supplementary file

## Data Availability

The datasets generated during and/or analysed during the current study are available from the corresponding author.
