# Supplementary material for: Changes in grassland management and linear infrastructures associated to the decline of an endangered bird population
Source: Sci Rep. 2020 Sep 16;10:15150. doi: 10.1038/s41598-020-72154-9 (PMC7495444; doi:10.1038/s41598-020-72154-9)
Supplement: Supplementary file 1 — Supplementary file [file 41598_2020_72154_MOESM1_ESM.pdf]

Changes in grassland management and linear infrastructures associated to the decline of an endangered bird population

Ana Teresa Marques*, Francisco Moreira, Rita Alcazar, Ana Delgado, Carlos Godinho, Hugo Sampaio, Pedro Rocha, Nuno Sequeira, Jorge M. Palmeirim & João Paulo Silva

DOI: 10.1038/s41598-020-72154-9

* Corresponding author: Ana Teresa Marques (ateresamarques@gmail.com). cE3c - Centro de Ecologia, Evolução e Alterações Ambientais, Faculdade de Ciências, Universidade de Lisboa, Edifício C2, Campo Grande, 1749-016 Lisboa, Portugal

Supplementary Information

Table S1 – Summary statistics for the GLMM models analyzing changes in little bustard density and our predictors (breeding habitat availability, stocking rate, cattle proportion, roads, power lines) across the two surveys (2003-2006 and 2016).

**Model coeficients**	Estimate	SE	t	p-value
**GLMM1: little bustard density**				
Intercept	2.680	0.334	8.02	0.000
Survey 2016	-1.239	0.301	-4.12	0.000
**GLMM2: breeding habitat availability**				
Intercept	0.567	0.035	16.23	0.000
Survey 2016	-0.116	0.027	-4.25	0.000
**GLMM3: stocking rate**				
Intercept	0.805	0.083	9.745	0.000
Survey 2016	0.163	0.056	2.921	0.005
**GLMM4: cattle proportion**				
Intercept	0.598	0.024	24.99	0.000
Survey 2016	0.157	0.015	10.44	0.000
**GLMM5: roads**				
Intercept	8.507	1.359	6.258	0.000
Survey 2016	1.660	0.458	3.625	0.000
**GLMM6: power lines**				
Intercept	0.425	0.040	10.632	0.000
Survey 2016	0.067	0.013	5.286	0.000

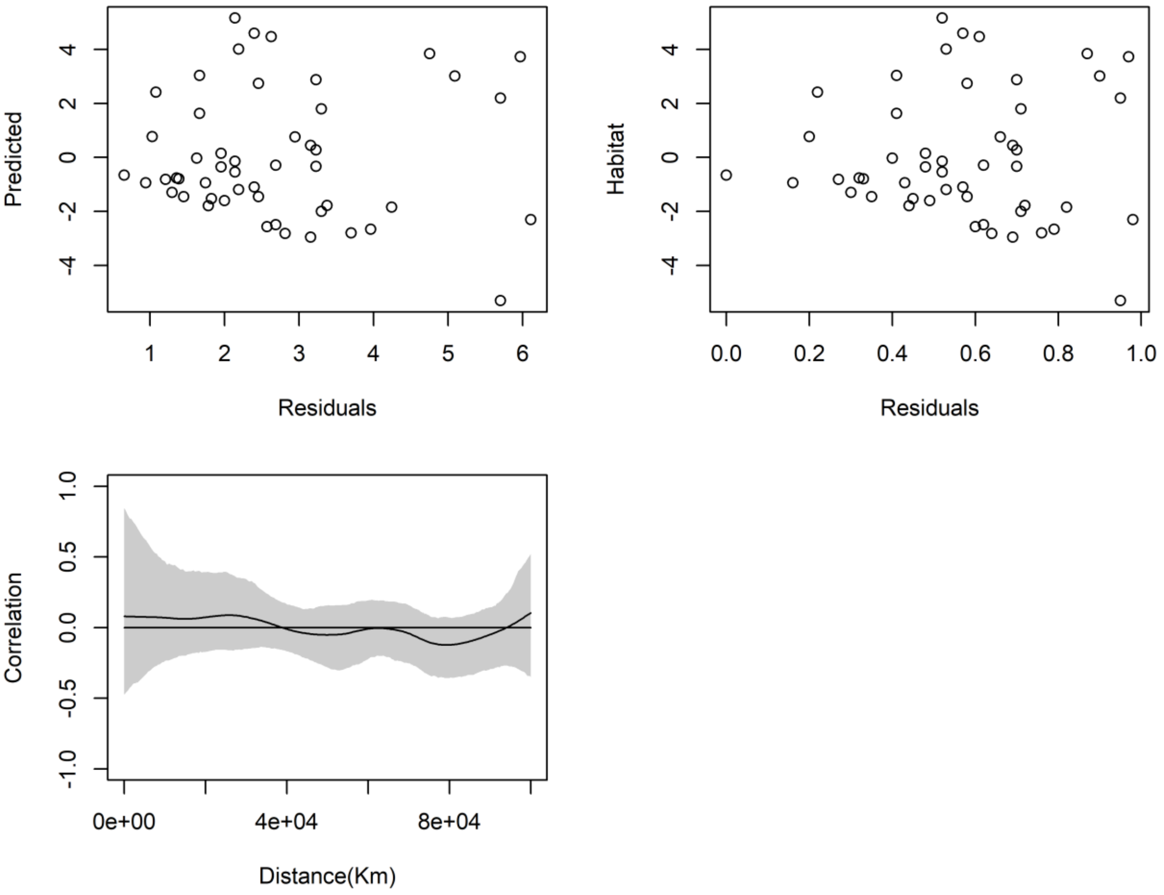

Figure S1 – Validation plots for the spatial model of the survey 2003-2006: residuals versus fitted values and residuals versus explanatory variables. A Spline correlogram describing the spatial autocorrelation in the residuals is presented in the bottom right corner. Lines represent the estimate (in the middle) and the 95% confidence envelopes (grey shadow) using 1000 bootstrap resamples[1].

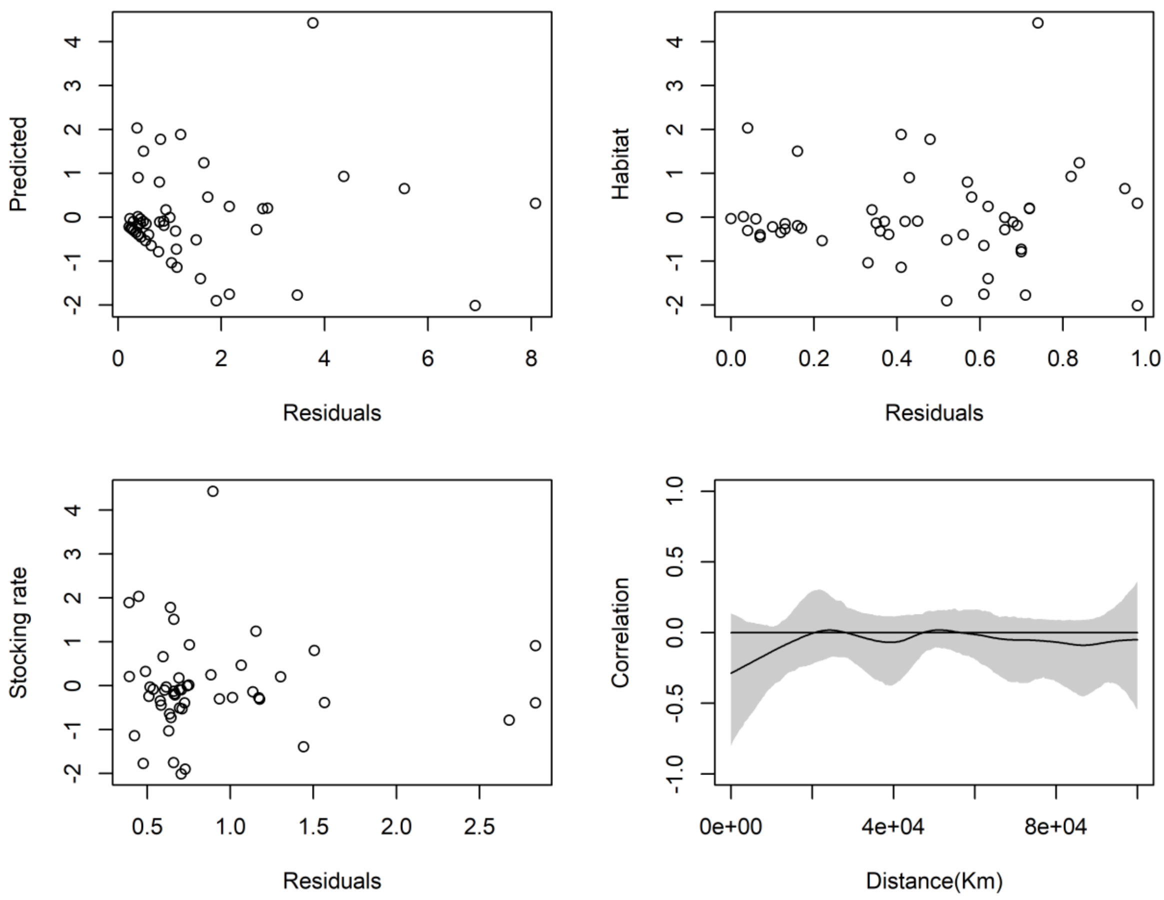

Figure S2 – Validation plots for the spatial model of the survey 2016: residuals versus fitted values and residuals versus explanatory variables. A Spline correlogram describing the spatial autocorrelation in the residuals is presented in the bottom right corner. Lines represent the estimate (in the middle) and the 95% confidence envelopes (grey shadow) using 1000 bootstrap resamples[1].

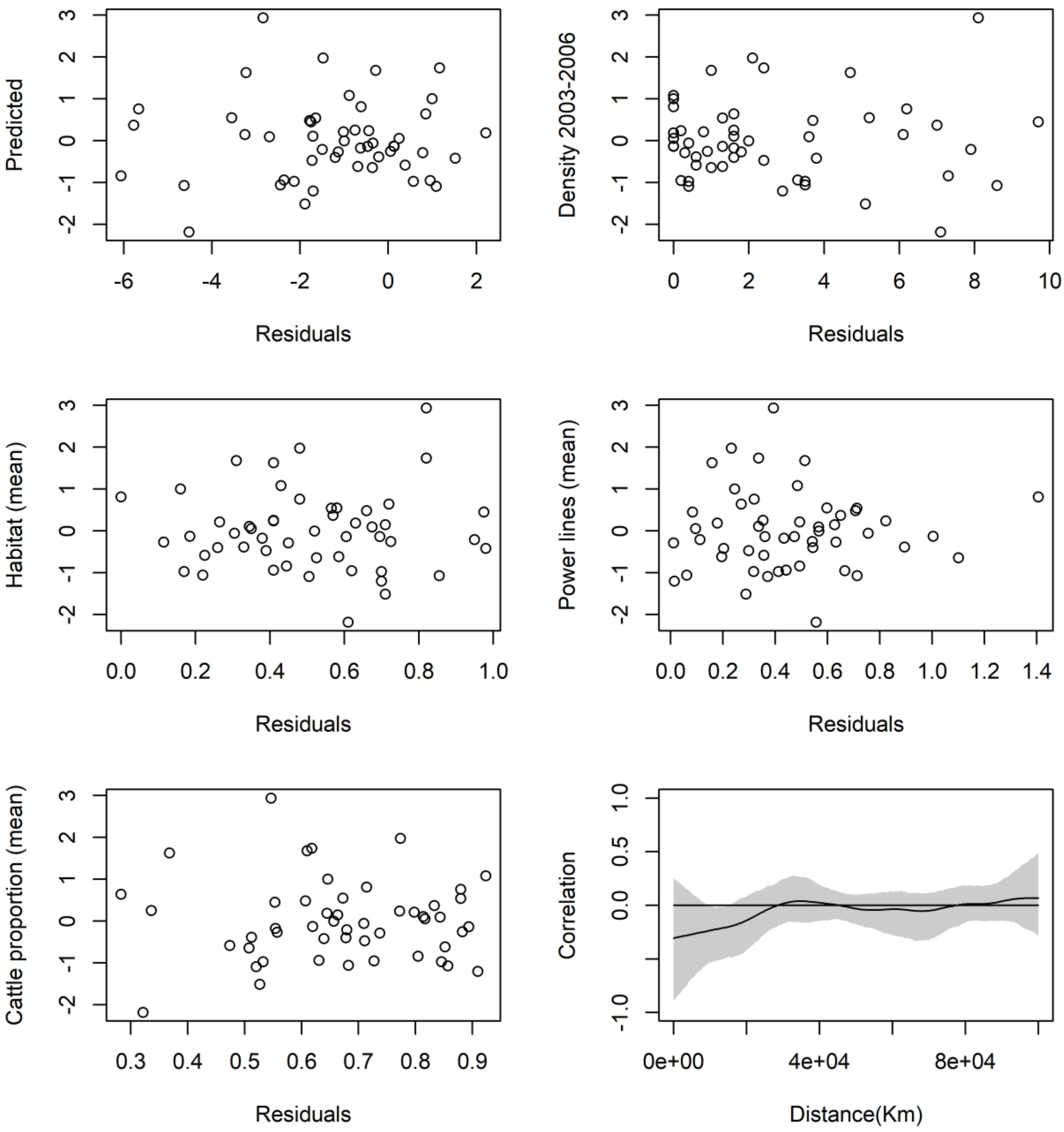

Figure S3 – Validation plots for the population variation model: residuals versus fitted values and residuals versus explanatory variables. A Spline correlogram describing the spatial autocorrelation in the residuals is presented in the bottom right corner. Lines represent the estimate (in the middle) and the 95% confidence envelopes (grey shadow) using 1000 bootstrap resamples[1].

a)

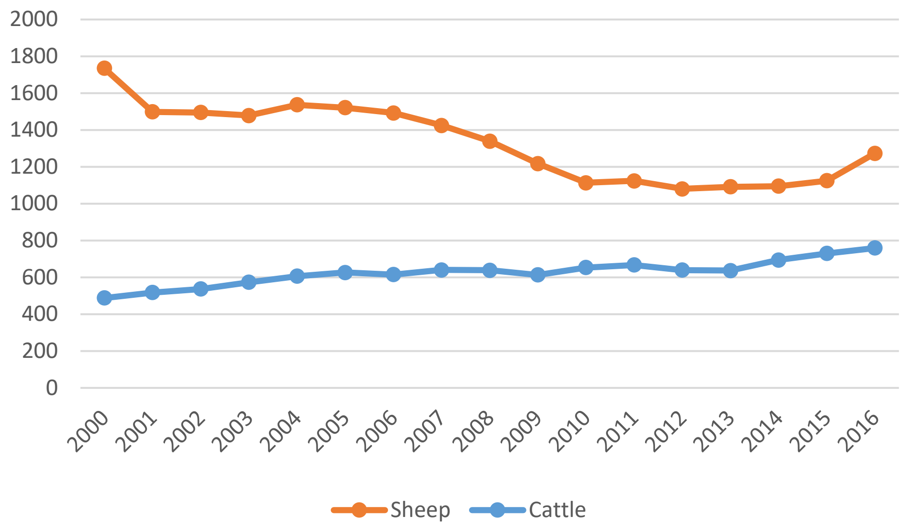

b)

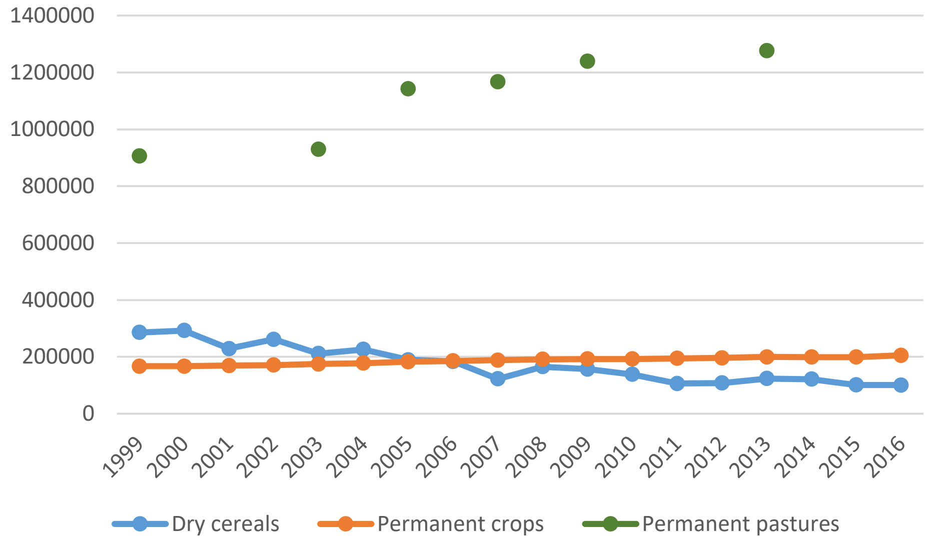

Figure S4 – Agricultural trends in Alentejo from 1999/2000 to 2016: a) sheep and cattle beef (number of animals), and b) dry cereals, permanent crops and permanent pastures (ha)[2].

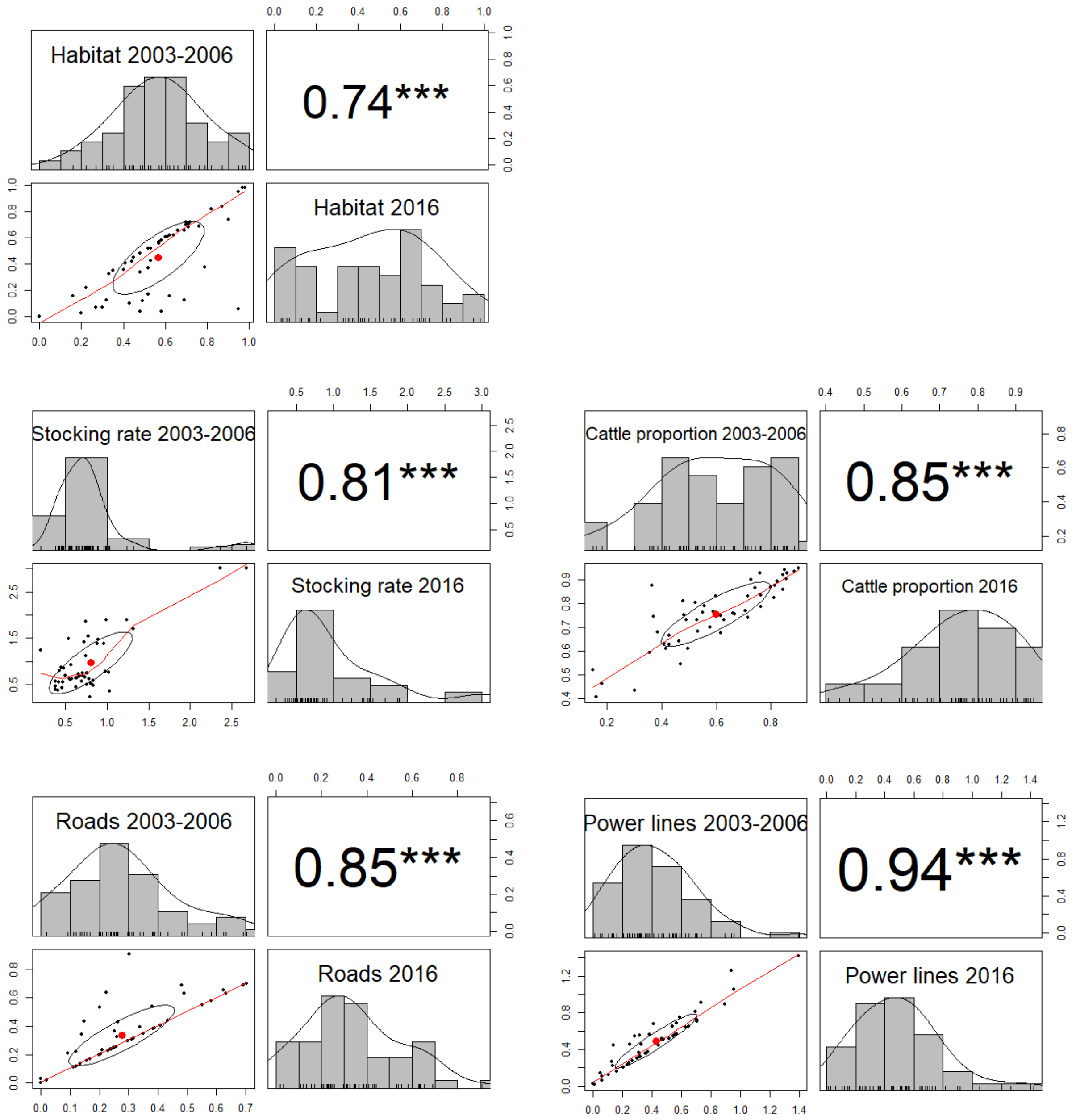

Figure S5 – Relations between predictors values measured across little bustard surveys, Bivariate scatter plots below the diagonal, histograms on the diagonal, and the Spearman's rank correlation coefficient (Spearman's rho) and p-value above the diagonal, as calculated by *pairs.panels* function from the psych package in R[3].

References:

1. Bjørnstad, O. N. & Falck, W. Nonparametric spatial covariance functions: Estimation and testing. *Environ. Ecol. Stat.* **8,** 53–70 (2001).
2. INE. Statistical data: Database. (2019). Available at: https://www.ine.pt/xportal/xmain?xpid=INE&xpgid=ine_base_dados. (Accessed: 9th May 2019)
3. Revelle, W. psych: Procedures for Psychological, Psychometric, and Personality Research. Northwestern University, Evanston, Illinois. R package version 1.9.12. (2019).
